# The Antioxidant Potential of Various Wheat Crusts Correlates with AGE Content Independently of Acrylamide

**DOI:** 10.3390/foods12244399

**Published:** 2023-12-07

**Authors:** Kristin Wächter, Carl Friedrich H. Longin, Patrick R. Winterhalter, Ute Bertsche, Gábor Szabó, Andreas Simm

**Affiliations:** 1 Department for Cardiac Surgery, University Hospital Halle (Saale), Martin-Luther University Halle-Wittenberg, 06120 Halle (Saale), Germany; patrick.winterhalter@uk-halle.de (P.R.W.); gabor.szabo@uk-halle.de (G.S.); andreas.simm@uk-halle.de (A.S.); 2 State Plant Breeding Institute, University of Hohenheim, 70599 Stuttgart, Germany; friedrich.longin@uni-hohenheim.de; 3 Core Facility Hohenheim, Mass Spectrometry Module, University of Hohenheim, 70599 Stuttgart, Germany; bertsche.ute@uni-hohenheim.de; 4 Center for Medical Basic Research, Martin-Luther University Halle-Wittenberg, 06120 Halle (Saale), Germany

**Keywords:** whole grain, antioxidants, advanced glycation end products, bread crust, acrylamide, wheat cultivars, cardioprotection

## Abstract

Epidemiological studies have indicated that the consumption of whole-grain products is associated with a reduced risk of cardiovascular diseases, type II diabetes, and cancer. In the case of bread, high amounts of antioxidants and advanced glycation end products (AGEs) are formed during baking by the Maillard reaction in the bread crust; however, the formation of potentially harmful compounds such as acrylamide also occurs. This study investigated the antioxidant responses of different soluble extracts from whole-grain wheat bread crust extracts (WBCEs) in the context of the asparagine, AGE, and acrylamide content. For that, we analyzed nine bread wheat cultivars grown at three different locations in Germany (Hohenheim, Eckartsweier, and Oberer Lindenhof). We determined the asparagine content in the flour of the 27 wheat cultivars and the acrylamide content in the crust, and measured the antioxidant potential using the induced expression of the antioxidant genes GCLM and HMOX1 in HeLa cells. Our study uncovered, for the first time, that the wheat crust’s antioxidant potential correlates with the AGE content, but not with the acrylamide content. Mass spectrometric analyses of WBCEs for identifying AGE-modified proteins relevant to the antioxidant potential were unsuccessful. However, we did identify the wheat cultivars with a high antioxidant potential while forming less acrylamide, such as Glaucus and Lear. Our findings indicate that the security of BCEs with antioxidative and cardioprotective potential can be improved by choosing the right wheat variety.

## 1. Introduction

The Maillard reaction, as a nonenzymatic browning reaction, takes place between protein amino groups and reducing sugars during heat processing, and results in the formation of advanced glycation end products (AGEs) as well as acrylamide [1,2,3]. While the health effects of AGEs have been controversially discussed from protective (antioxidant-active) to toxic [4], acrylamide is a clear carcinogenic compound [5]. On the one hand, AGE-rich bread crust extracts (BCEs) can induce NRF2 signaling and downstream targets such as HMOX1 and GCLM in endothelial cells, resulting in protection against oxidative stress [6]. In addition, BCEs have been shown to protect vascular grafts from in vitro ischemia/reperfusion injury [7]. Furthermore, feeding mice with rye bread crust induces antioxidant genes and suppresses the NF–κB pathway [8]. On the other hand, much research has been conducted to reduce the acrylamide formation in foods, especially in bread [9]. The common ways to mitigate acrylamide formation are heat reduction, fermentation prolongation, and additives [10]. For example, Hedegaard et al. reported that adding a 1% aqueous rosemary extract reduced the acrylamide content in wheat buns by 62% [11]. Furthermore, studies on a starch coating showed a significant reduction in the acrylamide content in bread crust [12]. Additionally, bread treated with a Tartary buckwheat sprout extract led to a 27.3% reduced acrylamide concentration [13]. During the baking process, asparagine is the responsible precursor for acrylamide formation [14,15]. Therefore, asparaginase can be added to inhibit acrylamide formation [10]. Another approach is plant breeding to reduce the asparagine content already in the raw material [16], combined with the genetic control of the grain amino acid composition [17]. Furthermore, in agricultural practice, a sulfur deficiency in the soil should be avoided, as it leads to increased asparagine levels in the grain [18]. Hence, the genetics of the wheat, the growing conditions, and the baking conditions can affect the products during baking. We therefore analyzed nine bread wheat cultivars, which were grown at three different locations, for asparagine content. Furthermore, the objective of this study was to determine the antioxidant potential and the acrylamide and AGE content of the wheat bread crust. We further analyzed the association between these parameters and found that the wheat crust’s antioxidant potential correlates with the AGE content independently of acrylamide.

## 2. Materials and Methods

### 2.1. Plant Material

The plant material consisted of nine different wheat cultivars originating from Germany. The field experiments were conducted at three different locations (Hohenheim—HOH, 48°43′07.3″ N 9°11′08.7″ E, altitude of 403 m, Germany; Oberer Lindenhof—OLI, 48°28′19.0″ N 9°18′29.3″ E, altitude of 700 m, Germany; and Eckartsweier—EWE 48°32′52.4″ N 7°52′32.5″ E, altitude of 140 m, Germany) in the 2018 season (Table 1). Cropping was performed similarly at all locations, with an intensive conventional farming practice and the application of about 200 kg of nitrogen per ha through the use of fertilizers, taking Nmin into account. According to best farming practices, the field plots were also treated with herbicides, growth regulators, and fungicides. All the field plots were machine-planted and -harvested. The harvested samples from each plot were cleaned with the haldrup seed cleaner LT-21 (HALDRUP GmbH, Ilshofen, Germany).

### 2.2. Bread Crust and Bread Crust Extracts

The wheat material was ground using a grain mill (Elsässer Getreidemühle, SAMAP ECOSYSTEME, Colmar, France). A dough was prepared from 1000 g of wheat flour, 600 mL of water, 5% baker’s yeast, and 2% NaCl using a dough-kneading machine (LEA25 2G, MANZ Backtechnik GmbH, Creglingen-Münster, Germany). The whole-grain wheat bread was baked (KornLiebchen, Halle (Saale), Germany; Table 2 shows the baking conditions) in a multideck oven (Friedrich Bäckerei Zubehör GmbH, Solingen, Germany). The crust was isolated from the bread using a four-edge grater (GEFU, Eslohe, Germany), ground in a mill at 8000 rpm (Retsch, ZM200, Verder Scientific, Birmingham, UK), and frozen at −20 °C.

Soluble wheat bread crust extracts (WBCEs) were generated as described in detail by Wächter et al. [6].

### 2.3. Cell Culture

HeLa cells (obtained from ATCC^®^CCL-2, passage No. 15–20; ATCC, Manassas, VA, USA) were cultured in Dulbecco’s Modified Eagle Medium; 4.5 g/L glucose (DMEM; Thermo Fisher Scientific, Waltham, MA, USA) containing 10% FCS and 100 U/mL penicillin and 100 µg/mL streptomycin in a 37 °C humidified incubator in the presence of 10% CO_2_. Cells (3 × 10^5^ cells per well) were seeded on 6-well dishes and were incubated with 13% WBCE for 24 h.

### 2.4. RNA Extraction, Reverse-Transcription, and Quantitative Real-Time Polymerase Chain Reaction (qRT-PCR)

The total RNA was isolated from cells by TRIzol extraction, reverse transcribed according to the protocol of iScript Advanced cDNA Synthesis Kit (Bio-Rad, Feldkirchen, Germany), and qRT-PCR was performed as described in Wächter et al. [6]. The mRNA expression levels of the genes GCLM and HMOX1 were normalized by the levels of internal reference gene YBX1 (Y-box binding protein 1) and calculated according to the DDCt method (qRT-PCR primers are listed in Wächter et al. [6]). Data were analyzed from three and four independent experiments. 

### 2.5. Statistical Analysis

The data are presented as mean values with standard deviation, and *n* represents the number of independent experiments. For statistical analysis of the data, the 2-tailed, unpaired Student’s *t*-test was applied. Results with *p* < 0.05 were considered statistically significant (* *p* < 0.05; ** *p* < 0.01; *** *p* < 0.001).

### 2.6. AGE-Fluorescence Measurements

The diluted WBCEs (1:50, in PBS) were excited at 360 nm and the AGE-associated fluorescence emission was detected at 440 nm in a 96-well microtiter plate (black, U-shape, Eppendorf, Germany) with the FLUOstar OPTIMA device (BMG LABTECH, Ortenberg, Germany). The data are presented as mean values with standard deviation from three independent measurements.

### 2.7. Determination of Asparagine Content

The asparagine content of the wheat flour was determined in mg/kg according to the European Commission Regulation (EC No 152/2009, Annex III, Method F) with moderate modifications as reported in Rapp et al. [16].

### 2.8. Determination of Acrylamide Content by LC-ESI-MS/MS Analysis

#### 2.8.1. Sample Preparation for LC-ESI-MS/MS Analysis

One gram bread crust powder was spiked with 200 µL internal standard (d3-acrylamide, Sigma-Aldrich, St. Louis, MO, USA, 5 µg/mL in methanol) and extracted with 20 mL water using an ultrasonic bath (SONOREX Digitec DT, Bandelin, Berlin, Germany) for 12 min at 40 °C. Samples were centrifuged at 2500× *g* for 5 min and the supernatant was filtered through a filter paper (3HW, Sartorius AG, Goettingen, Germany). Proteins were precipitated by adding methanol to a ratio of methanol:water 80:20 (*v*/*v*) and incubation for 90 min at −20 °C. Then, samples were centrifuged at 2500× *g* and the supernatant was filtered through a filter paper (3HW, Sartorius AG, Goettingen, Germany). The filtrate was dried using an XcelVap concentrator (Biotage, Uppsala, Sweden). The samples were resuspended in 10 mL water and further purified by SPE cartridges (CHROMABOND ABC18, 45 µm, 6 mL/500 mg, Machery & Nagel, Düren, Germany). The SPE cartridges were activated with 10 mL methanol and then rinsed with 10 mL water. The samples (10 mL) were loaded on the SPE cartridges and the first two milliliters were discarded, while the rest of the flow through was collected and concentrated ten-fold using an XcelVap concentrator. 

#### 2.8.2. LC-ESI-MS/MS Analysis

Mass spectrometry analysis of the acrylamide content in bread crusts was performed by high-performance liquid chromatography electrospray tandem mass spectrometry (HPLC-ESI-MS/MS) using a 1290 Infinity HPLC system (Agilent, Waldbronn, Germany) coupled to a 5500 QTRAP^®^ mass spectrometer (SCIEX, Framingham, MA, USA) equipped with a TurboV electrospray ion source. The QTRAP mass spectrometer was operated in positive electrospray ionization mode and the acrylamide was detected using multiple reaction monitoring (MRM). MRM transitions *m*/*z* 72 > 55 (quantifier) and 72 > 27 were used for the identification and the quantification of the acrylamide, utilizing collision energies of 15 and 29 eV, respectively. The d3-acrylamide internal standard was monitored by MRM transitions *m*/*z* 75 > 75, 75 > 58 (quantifier), and 75 > 30, utilizing collision energies of 10, 22, and 35 eV, respectively. The dwell time for each MRM transition was 450 ms. The following general mass spectrometry settings were used: electrospray voltage 5500 V, curtain gas 35 (arbitrary units), Ion Source One and Two 70 and 30 (arbitrary units), and probe temperature 350 °C. HPLC separation was performed on a Luna Omega Polar C18, 150 mm × 2.1 mm column (particle size 1.6 µm, pore size 100 Å, Phenomenex, Germany) at a flow rate of 350 µL/min and a column temperature of 30 °C. The mobile phase consisted of 0.1% formic acid (FA, solvent A) and methanol, 0.1% FA (solvent B). The following gradient conditions were used: 0 min 3% B, 0–1.5 min 5% B, 1.5–2 min 5% B, 2–4 min 20% B, 4–5 min 100% B, 5–8 min 100% B, 8–9 min 3% B, and re-equilibration at 3% B for 5 min. Total run time was 15 min. The data acquisition and quantification was performed using Analyst 1.7.2 software (SCIEX, Framingham, MA, USA).

### 2.9. Mass Spectrometric Analyses of the Soluble WBCEs

#### 2.9.1. Protein Extraction for Comparative Proteome Analysis

The soluble wheat bread crust extracts (WBCEs) were precipitated using chloroform-methanol precipitation [19]. The protein pellets were resuspended in 6 M urea in 50 mM Tris-HCl (pH 8.5), and the protein concentrations were determined by the Bradford assay [20].

#### 2.9.2. In-Solution Digest of Proteins and Peptide Purification

A total of 10 µg of the wheat protein extract in 60 µL 6 M urea, 50 mM Tris HCl (pH 8.5) was used for the in-solution digests. DTT was added to a final concentration of 10 mM for the reduction of cysteines. The samples were incubated for 30 min at 56 °C under shaking at 1000 rpm in an Eppendorf Thermomixer. Alkylation of cysteines was performed by adding 30 mM iodoacetamide and incubation for 45 min at room temperature in the dark. Alkylation was stopped by adding 50 mM DTT and samples were incubated for another 10 min at RT. Then, 500 ng LysC protease (Roche, Basel, Schweiz) in 50 mM Tris HCl (pH 8.5) was added and the samples were digested overnight at 30 °C. Next, the urea in the reaction mixture was diluted to 2 M by adding the appropriate amount of 50 mM Tris HCl (pH 8.5). One µg trypsin (Roche, Basel, Schweiz) in 50 mM Tris HCl (pH 8.5) was added and the digestion was continued for 4 h at 37 °C. The digest was stopped by adding 3 µL 10% TFA (trifluoroacetic acid). Next, the peptide mixtures were concentrated and desalted using C18 stage tips [21] and dried under vacuum. The dried samples were dissolved in 30 µL 0.1% TFA. Aliquots of 5 µL were subjected to nanoLC-MS/MS analysis. 

#### 2.9.3. Mass Spectrometry Analysis

The nanoLC-ESI-MS/MS experiments were performed on an UltiMate 3000 RSLCnano system (Thermo Fisher Scientific, Waltham, MA, USA) coupled to a Q-Exactive HF-X mass spectrometer (Thermo Fisher Scientific, Waltham, MA, USA) using a Nanospray Flex ion source (Thermo Fisher Scientific, Waltham, MA, USA). The tryptic digests were concentrated and desalted on a precolumn (300 µm × 5 mm, Acclaim PepMap100 C18, 5 µm particle size, 100 Å pore size) and separated on a nanoEase MZ HSS T3 analytical column (25 cm × 75 μm, 1.8 µm particle size, 100 Å pore size, Waters, Milford, MA, USA) operated at constant temperature of 35 °C. The peptides were separated at a flow rate of 300 nL/min using a 90 min gradient with the following profile: 2–55% solvent B in 90 min, 55–95% solvent B in 5 min, and maintained at 90% solvent B for 5 min (0.5% acetic acid (solvent A) and 0.5% acetic acid in acetonitrile/H_2_O (80/20, *v*/*v*, solvent B) were used as solvents). 

The Q Exactive HF-X was operated under the control of XCalibur 4.3.73 software. MS spectra (*m*/*z* = 300–1800) were detected in the Orbitrap at a resolution of 60,000 (at *m*/*z* = 200) using a maximum injection time (MIT) of 100 ms and an automatic gain control (AGC) value of 1 × 10^6^. The internal calibration of the Orbitrap analyzer was performed using lock-mass ions from ambient air, as described in Olsen et al. [22]. Data-dependent MS/MS spectra were generated for the 30 most abundant peptide precursors in the Orbitrap using higher-energy C-trap dissociation (HCD) fragmentation at a resolution of 15,000, a normalized collision energy of 27, and an intensity threshold of 1.6 × 10^5^. Only ions with charge states from +2 to +5 were selected for fragmentation using an isolation width of 1.6 Da. For each MS/MS scan, the AGC was set at 2 × 10^5^ and the MIT was 50 ms. Fragmented precursor ions were dynamically excluded for 30 s within a 5 ppm mass window to avoid repeated fragmentation. 

#### 2.9.4. Data Analysis

The MS data were analyzed by MaxQuant (version 1.6.17.0) [23] and Andromeda search engine [24] with standard settings except noted otherwise. The UniProt triticum aestivum-database was used (downloaded on 2 June 2022, unreviewed database entries: 130312). The carbamidomethylation of cysteine was set as a fixed modification. A maximum of two modifications per peptide were allowed. To identify interesting masses associated with AGEs that could be further analyzed by variable modification setup, the “Dependent peptide” search was activated. Variable modifications were set on lysine (K) or arginine (R): Hex (KR) 162.05282 Da, MG-H1/2/3 (R) 54.01056 Da, MGH-DH or CEA (R) 72.02113 Da, 3-DG or THP (R) 144.04226 Da. A minimal peptide length of seven amino acids and a maximum of three miscleavages (Trypsin/p) were allowed. The maximum protein, peptide, and site false discovery rates (FDR) were set to 0.01. “Re-quantify” and “Match between runs (2 min)” was activated. “Second peptides” and “Use .NET Core” was deactivated. The default values for label-free quantification (LFQ) were taken. For the first search, the reviewed database UniProt triticum aestivum-database was used (downloaded on 13 May 2022, entries: 361).

The automatic bioinformatics data analysis was carried out with Perseus (version 1.6.14.0) [25]. Only identified by site, reverse, and potential contaminant were rejected. For the modification analysis, the mod/base ratio of at least 5 of 27 samples was bigger than zero. For the protein analysis, only intensities of LFQ-proteins of all 27 samples were allowed. For each AGE modification, a ranking for all modified peptides was performed for the 27 samples (rank 27 for best mod/base ratio; rank 1 for not or least mod/base ratio) and the mean value of all modified peptide ranks was calculated for each sample).

## 3. Results

### 3.1. Analysis of Wheat Flour and Wheat Bread Crust Extracts (WBCEs)

#### 3.1.1. AGE-Related Fluorescence

**Figure 1:**
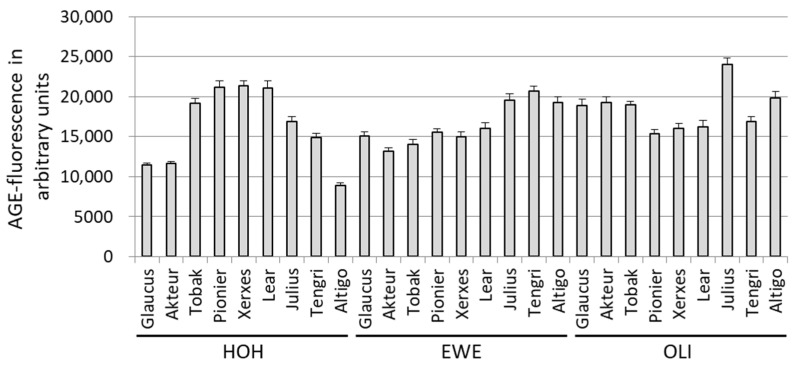
AGE-related fluorescence measured for nine wheat cultivars grown at three different locations (HOH (Hohenheim), EWE (Eckartsweier), and OLI (Oberer Lindenhof)). The data are presented as mean values with standard deviation from three independent measurements (technical replicates).

Nine wheat cultivars were grown at three different locations in Germany (Hohenheim, Eckartsweier, Oberer Lindenhof), i.e., 27 batches of flour were baked with the same recipe (Supplemental Figure S1). The soluble extracts were obtained from these bread crusts, and AGE-related fluorescence was measured (Figure 1). The AGE-related fluorescence acted as a measure for AGE formation during the baking process, and varied in the different cultivars. The wheat cultivar Julius, grown in Oberer Lindenhof, showed the strongest signal. In addition, the AGE-related fluorescence in one wheat cultivar was not the same at all locations. No significant differences between the wheat cultivars or the locations were observed for the AGE-related fluorescence (Supplemental Figure S2a,b).

#### 3.1.2. Antioxidant Potential of Soluble WBCEs

**Figure 2:**
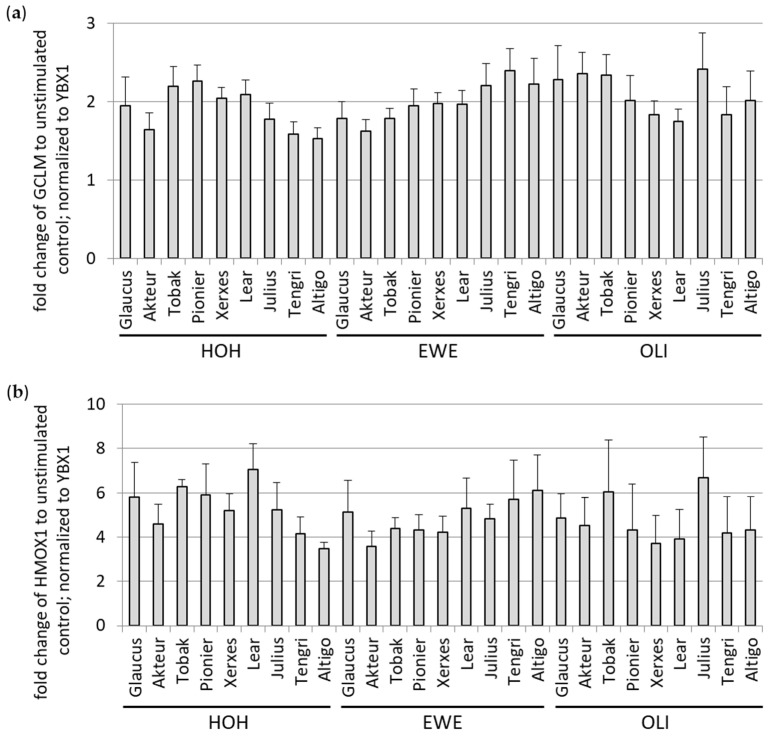
The antioxidant potential of the soluble WBCEs. Upon incubation of HeLa cells with WBCEs, the mRNA abundance of the antioxidant gene GCLM (**a**) and HMOX1 (**b**) was measured by qRT-PCR and was normalized to reference gene YBX1. Data were analyzed from four (**a**) and three (**b**) independent experiments and were depicted as means with standard deviation. Mounting place is indicated by HOH (Hohenheim), EWE (Eckartsweier), and OLI (Oberer Lindenhof) together with the names of the nine wheat cultivars.

In a previous study with soluble extracts from rye-wheat mixed bread crust (BCE), we showed an induction of the antioxidant gene expression in cells treated with BCE [6]. In order to analyze the antioxidant potential of the different soluble WBCEs, HeLa cells were stimulated for 24 h with WBCEs. Thereafter, RNA was isolated, and two genes encoding for antioxidant enzymes, namely, GCLM and HMOX1, were analyzed by qRT-PCR (Figure 2). The antioxidant potential of the various extracts was heterogeneous; however, both transcripts were uniformly regulated in their expression (Supplemental Figure S3). No significant differences between the wheat cultivars or the locations were observed for GCLM and HMOX1 (Supplemental Figures S4a,b and S5a,b).

#### 3.1.3. Determination of Asparagine and Acrylamide

**Figure 3:**
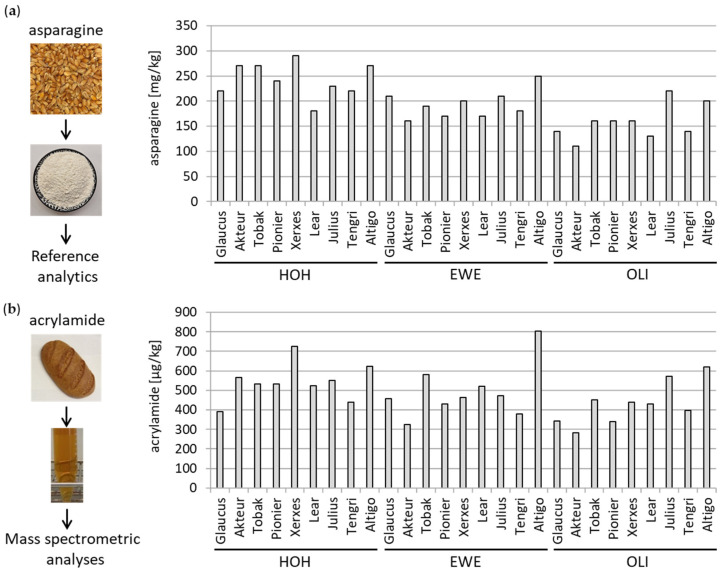
Determination of asparagine and acrylamide. Asparagine content was determined in the flour in mg/kg (**a**). Acrylamide content was measured in water-soluble extracts of the 27 wheat crusts by mass spectrometry in µg/kg (**b**). Mounting place is indicated by HOH (Hohenheim), EWE (Eckartsweier), and OLI (Oberer Lindenhof) together with the names of the nine wheat cultivars.

The asparagine amount, as an acrylamide precursor, was determined in the different wheat cultivars (Figure 3a). Upon baking, the acrylamide level were determined in the soluble WBCEs by mass spectrometry (Figure 3b). It is noteworthy that wheat cultivars grown in Oberer Lindenhof (OLI) had a significantly lower asparagine content in the flour compared to wheat cultivars grown in Hohenheim (Supplemental Figure S6a). Similarly, the acrylamide content was significantly lower in the bread crust of the wheat cultivars grown at Oberer Lindenhof compared to Hohenheim (Supplemental Figure S6b). Furthermore, the wheat cultivar Lear had a significantly lower asparagine level compared to the wheat cultivar Julius and Altigo (Supplemental Figure S7). Consistently, the wheat cultivar Lear had a significant lower acrylamide level compared to wheat cultivar Altigo (Supplemental Figure S8). Significantly lower acrylamide levels were also measured for the wheat cultivar Glaucus in comparison to the wheat cultivar Julius and Altigo, for the wheat cultivar Pionier in comparison to Altigo, and for the wheat cultivar Tengri in comparison to Julius and Altigo (Supplemental Figure S8). 

### 3.2. Association between Different Parameters of Wheat Flour and WBCEs

**Figure 4:**
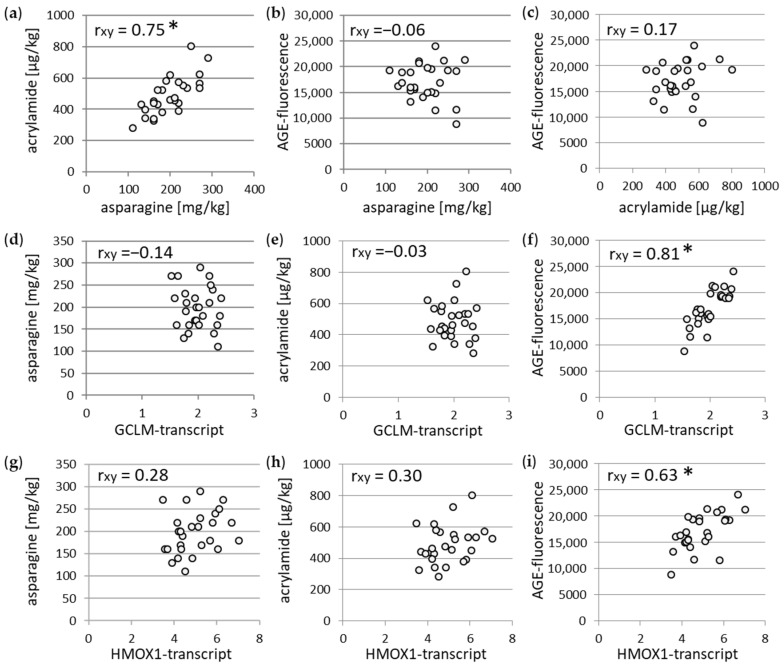
Association between different parameters was determined by Pearson correlation coefficient (r_xy_), for acrylamide and asparagine (**a**), AGE-fluorescence and asparagine (**b**), AGE-fluorescence and acrylamide (**c**), asparagine and GCLM-transcript (**d**), acrylamide and GCLM-transcript (**e**), AGE-fluorescence and GCLM-transcript (**f**), asparagine and HMOX1-transcript (**g**), acrylamide and HMOX1-transcript (**h**), and AGE-fluorescence and HMOX1-transcript (**i**). * *p* < 0.0001.

To further explore the interplay of the determined parameters in the wheat flour and the WBCE, correlation analyses were performed. In line with other studies, we found a strong and significant correlation between the asparagine level in the flour and the acrylamide content in the WBCEs (Figure 4a). In contrast, no correlation was observed between the AGE-fluorescence and the asparagine level as well as the acrylamide level (Figure 4b,c). Furthermore, the correlation analysis between the antioxidant transcript GCLM and the asparagine as well as the acrylamide revealed no association (Figure 4d,e). However, the GCLM-transcript and the AGE-fluorescence indicated a strong significant dependency (Figure 4f). Consistently, no association was found for the HMOX1-transcript and the asparagine as well as the acrylamide (Figure 4g,h), whereas a strong significant correlation was observed between the HMOX1-transcript and the AGE-fluorescence (Figure 4i).

### 3.3. Mass Spectrometric Analyses of WBCEs

**Figure 5:**
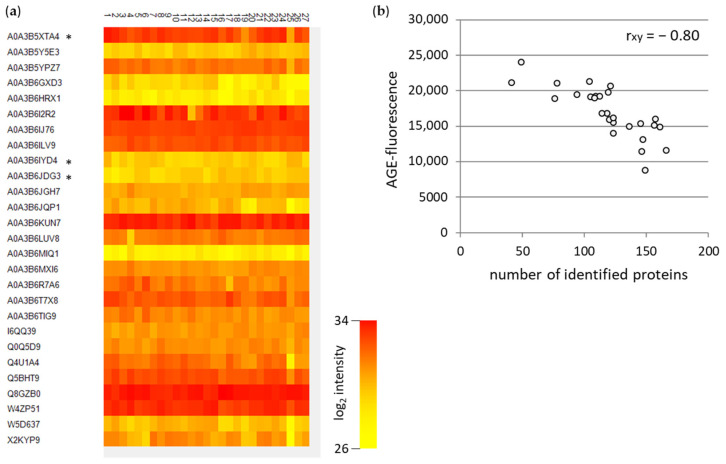
Mass spectrometric analyses of the soluble WBCEs. The heat map (**a**) exhibits the proteins found in all 27 samples. Marked candidates with the asterisk (*) are representative for multiple homologous proteins. (**b**) The association between the AGE-fluorescence and the number of identified proteins is determined by the Pearson correlation coefficient (r_xy_).

We further analyzed the soluble WBCEs by mass spectrometry. The abundance of the proteins found in all 27 samples is displayed in a heat map (Figure 5a). Very abundant proteins such as A0A3B6KUN7 are shown in red, while less abundant proteins such as A0A3B6MIQ1 are indicated in yellow. However, the correlation between the number of identified proteins and the AGE-fluorescence exhibited a negative relation (Figure 5b). This result indicates that the intensive baking leads to less protein identification. One reason could be that the AGE formation induces cross-linking and impedes the enzymatic digestion, thereby hampering the identification. Another reason for the weaker protein identification is the baking process itself, which promotes protein degradation.

#### 3.3.1. Mass Spectrometric Analyses of Specific AGE Modifications in WBCEs

**Figure 6:**
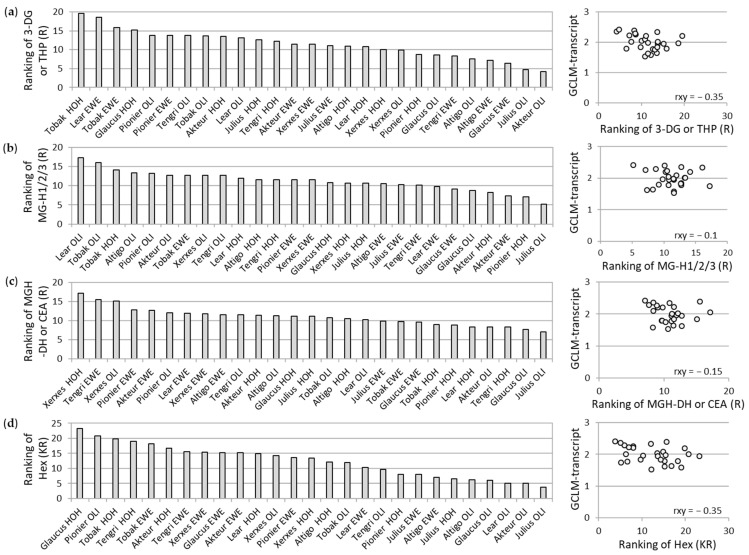
Analysis of different AGE modifications by mass spectrometry. The relative values of modified versus unmodified peptides are exhibited by ranking from 1–27 (for not or least abundant modifications to highly abundant modifications) for the additional mass of 3-DG or THP (**a**), MG-H1/2/3 (**b**), MGH-DH or CEA (**c**), and hexose (**d**). The association between GCLM-transcript and the ranking of the respective modification is determined by the Pearson correlation coefficient (r_xy_). The mounting place is indicated by HOH (Hohenheim), EWE (Eckartsweier), and OLI (Oberer Lindenhof) together with the names of the nine wheat cultivars.

Since we observed a correlation between the AGE-related fluorescence and the antioxidant potential of WBCEs, we performed mass spectrometric analyses to identify specific AGE modifications in the WBCEs (Figure 6). We determined relative modification values by ranking from 1–27 (one stands for not or least abundant modifications (ratio modified to nonmodified), whereas 27 stands for high abundant modifications). The following modifications were validated: 3-deoxyglucosone (3-DG) or mass identical tetrahydropyrimidine (THP) (Figure 6a), several forms of the methylglyoxal-derived hydroimidazolone (MG-H1/2/3) (Figure 6b), the dihydroxyimidazolidine (MGH-DH) or mass identical carboxyethylarginine (CEA) (Figure 6c), and the hexose as a pre-stage of AGEs (Figure 6d). In a second step, the association between the GCLM-transcript and the ranking of the respective modification was determined by the Pearson correlation coefficient (r_xy_). There was no correlation of the analyzed AGE modifications to the GCLM-transcript found. Nevertheless, the mean of all ranked AGE modifications, including hexose, exhibited an increase in the wheat cultivars Tobak and Xerxes (Supplemental Figure S9).

**Figure 7:**
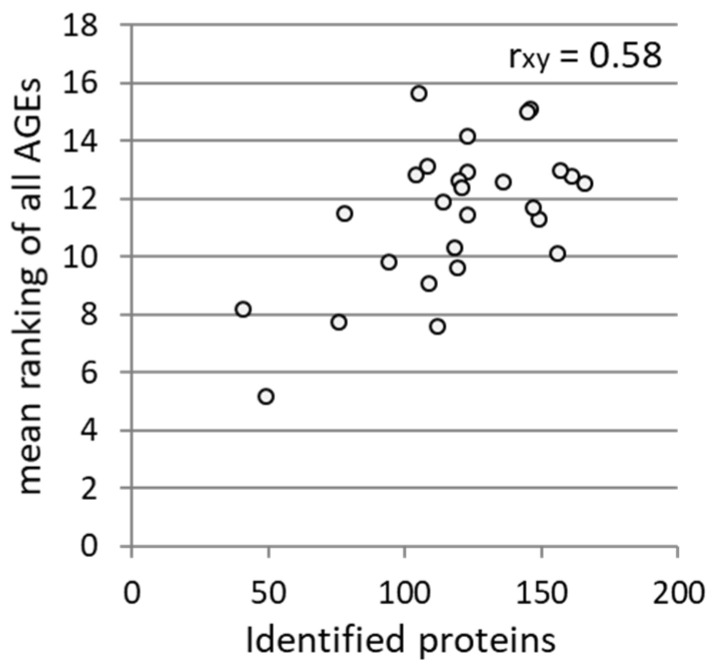
Association between mean ranking of all AGEs (Figure 6) and the number of the identified proteins, determined by the Pearson correlation coefficient (r_xy_).

Since the association between the mean ranking of all AGEs (mean value from all AGE ranks) and the number of identified proteins showed a good correlation (Figure 7), presumably the before-mentioned impeded enzymatic digestion by the AGE formation exists, leading to a decreased identification of the proteins as well as AGE modifications.

#### 3.3.2. Mass Spectrometric Analyses of AGE Modifications at Specific Peptides

**Figure 8:**
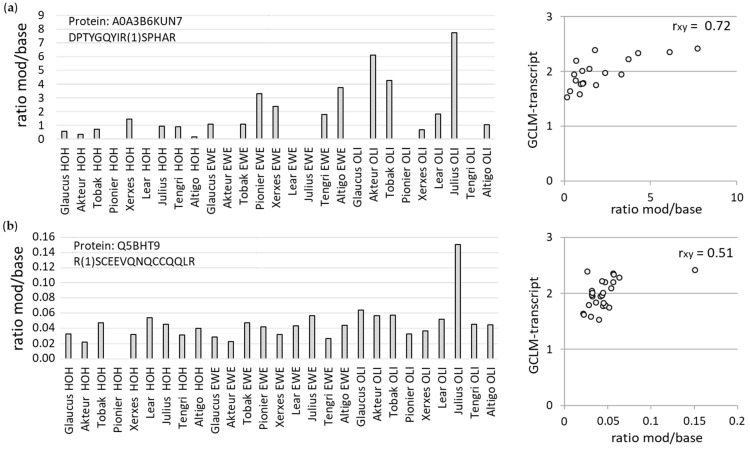
Ratio of the MG-H1/2/3 modified peptides to the unmodified peptides of protein A0A3B6KUN7 (**a**) and Q5BHT9 (**b**) among all 27 WBCEs. Association between the GCLM-transcript and the ratio mod/base for the modification at the indicated peptide was determined by the Pearson correlation coefficient (r_xy_).

Among the abundant proteins identified in all WBCEs, we found MG-H1/2/3 modified peptides, which correlated to the antioxidant potential among all 27 WBCEs (Figure 8). For example in A0A3B6KUN7, the MG-H1/2/3 modified peptide DPTYGQYIR(1)SPHAR exhibited a dependence on the antioxidant potential (Figure 8a), whereas in Q5BHT9, the MG-H1/2/3 modified peptide R(1)SCEEVQNQCCQQLR showed an association with the antioxidant potential (Figure 8b). Noteworthily, these specific modifications were found to be particularly frequently in the Julius wheat cultivar grown in Oberer Lindenhof, which had the highest AGE-related fluorescence (Figure 1).

## 4. Discussion

The present study investigated the antioxidant responses of different soluble extracts from whole-grain wheat bread crust in the context of further properties like the asparagine, which was measured in the flour, as well as the AGEs and the acrylamide, which were measured in the soluble bread crust extracts. Both AGEs and acrylamide arise within the Maillard reaction [2,26,27]. The Maillard reaction is also the source of the flavor and the aroma of heated foods, which consumers expect. Hence, the browning reaction is like a double-edged sword. Acrylamide is considered as a neurotoxic substance [28,29] and is classified as probably carcinogenic to humans (Group 2A carcinogen) by the IARC (International Agency for Research on Cancer) [30]. In line with previous studies [31,32] we could show that the acrylamide levels in the WBCEs strongly depend on the asparagine content of the flour. However, the antioxidant potential represented by the activation of the NRF2 downstream targets GCLM and HMOX1 exhibited only a correlation on the AGE-fluorescence but not on the acrylamide content of the bread crust, nor on the asparagine content of the whole-grain flour. In addition, we found that the WBCEs of the wheat cultivars Glaucus and Lear have a high antioxidant potential and form less acrylamide.

One limitation of this study is that not all AGEs exhibit fluorescent properties; thus, the measured fluorescence represents only AGE-compounds like pentosidine as fluorescent glycoxidation marker for AGEs [33,34]. A study by Rapp and colleagues, with 149 bread wheat cultivars grown at three locations, revealed that a reduction of 64% in asparagine is achievable through the choice of the wheat cultivar [16]. For our studied nine wheat cultivars grown at three locations, we also found for the cultivar Lear a significantly lower asparagine and acrylamide level. Glaucus, Pionier, and Tengri also showed a significantly lower acrylamide content. These data suggest that varieties that form less acrylamide could be selected across environments. We also observed an influence of the mounting place. Some studies reported that environmental factors might affect the asparagine content [35]. For instance, a lack of sulfur leads to an increased asparagine content [18], and processing of flour derived from sulfate-deprived wheat results in a strong acrylamide formation [36]. Studies by Claus and colleagues indicated that the increased acrylamide levels could be caused by nitrogen fertilization [37].

Attempts to identify responsible AGE modifications within the soluble extracts were not successful. With the standard tryptic digestion methods, AGE modifications can be barely resolved. This was reflected in the negative correlation between identified proteins by mass spectrometry and the AGE-fluorescence (Figure 5b). Trypsin cleaves C-terminal to arginine or lysine residues, which are probably masked by the AGE modifications. Furthermore, an increased temperature might promote the AGE-mediated interprotein cross-linking, as described for the α-crystallin-client proteins [38]. Consequently, the digestion of the proteins is further impaired. Another reason for the weaker protein identification is the baking process itself, which promotes protein degradation. Therefore, identifying AGE-modified peptides and proteins in strongly heated food products is challenging. Future research is necessary to improve the digestion strategies of AGE-modified proteins and their quantification by mass spectrometry.

The antioxidant potential of the WBCEs might also be mediated by phenolic compounds like flavonoids and phenolic acids [39]. A role in the acrylamide reduction was postulated for plant extracts and their antioxidant activity [11]. No relation was found in the correlation analyses between the acrylamide content and the antioxidant activity of the WBCEs. This might be at least partly due to the relatively weak antioxidant activity of the WBCEs. In comparison, BCE (bread crust extract of rye-wheat mixed bread) showed a more potent induction of NRF2 downstream targets [6]. Therefore, future studies in which extracts with antioxidant activity are added to the wheat dough may also help to curb the acrylamide development. For instance, adding green tea extracts reduced the acrylamide levels in fried chicken [40], and for apple extracts, respectable inhibitory effects on the acrylamide formation in fried potatoes were observed [41]. One mechanism by which antioxidants mitigate the harmful effects of acrylamide might be the direct destruction of the acrylamide by their corresponding oxidation products [42]. Studies in potato crisps during frying exhibited a positive correlation between the total antioxidant capacity (TAC) and the acrylamide formation, therefore the authors concluded that antioxidant compounds (like melanoidins) and acrylamide formed at similar stages of the Maillard reaction [43].

In summary, the present study showed that it is possible to develop antioxidant active WBCEs while containing less acrylamide. In addition, the reduction strategies for the acrylamide during baking should take into account risks and advantages, since the Maillard reaction is the essential source for taste and formation of antioxidant compounds. Whether the wheat bread crust with less acrylamide mediates antioxidant effects in vivo remains to be further investigated by animal feeding experiments and analyses of different tissues.

## Figures and Tables

**Table 1:** Wheat cultivars and their growth locations.

Wheat Cultivar	Growth Location
Glaucus	HOH, EWE, OLI
Akteur	HOH, EWE, OLI
Tobak	HOH, EWE, OLI
Pionier	HOH, EWE, OLI
Xerxes	HOH, EWE, OLI
Lear	HOH, EWE, OLI
Julius	HOH, EWE, OLI
Tengri	HOH, EWE, OLI
Altigo	HOH, EWE, OLI

**Table 2:** Baking conditions.

Grain	Dough Resting	Baking Time	Top Heat	Bottom Heat
wheat	30 min	50–60 min	250	230

## Data Availability

The data presented in this study are available on reasonable request from the corresponding author. The data are not publicly available due to privacy restrictions.

## Supplementary Materials

The following supporting information can be downloaded at: https://www.mdpi.com/article/10.3390/foods12244399/s1, Figure S1: Bread baking. Figure S2: Wheat cultivar and location dependence of AGE-fluorescence. Figure S3: The association between GCLM- and HMOX1-transcript. Figure S4: Wheat cultivar and location dependence of GCLM induction. Figure S5: Wheat cultivar and location dependence of HMOX1 induction. Figure S6: Location dependence of asparagine and acrylamide. Figure S7: The mean asparagine value of each cultivar grown at three different places. Figure S8: The mean acrylamide value of each cultivar grown at three different places. Figure S9: The mean of all ranked AGE modifications and hexose.

## Abbreviations

3-DG	3-deoxyglucosone
AGEs	advanced glycation end products
BCE	bread crust extract
CEA	carboxyethylarginine
EWE	Eckartsweier
FA	formic acid
FCS	fetal calf serum
GCLM	glutamate-cysteine ligase modifier subunit
HeLa	human cervical carcinoma
Hex	hexose
HMOX1	heme oxygenase 1
HOH	Hohenheim
HPLC	high performance liquid chromatography
HPLC-ESI-MS/MS	high performance liquid chromatography electrospray tandem mass spectrometry
LFQ	label-free quantification
MG-H1/2/3	methylglyoxal-derived hydroimidazolones
MGH-DH	dihydroxyimidazolidine
Mod	modified
MRM	multiple reaction monitoring
NRF2	nuclear factor E2-related factor 2
OLI	Oberer Lindenhof
PBS	phosphate-buffered saline
qRT-PCR	quantitative Real time-PCR
TFA	trifluoroacetic acid
THP	tetrahydropyrimidine
WBCE	soluble extract from whole-grain wheat bread crust
YBX1	Y-box binding protein 1
