# Supplementary material for: The Antioxidant Potential of Various Wheat Crusts Correlates with AGE Content Independently of Acrylamide
Source: Foods. 2023 Dec 7;12(24):4399. doi: 10.3390/foods12244399 (PMC10743060; doi:10.3390/foods12244399)
Supplement: Supplementary file 1 [file foods-12-04399-s001.zip › foods-2727791-supplementary.pdf]

Supplementary figures:

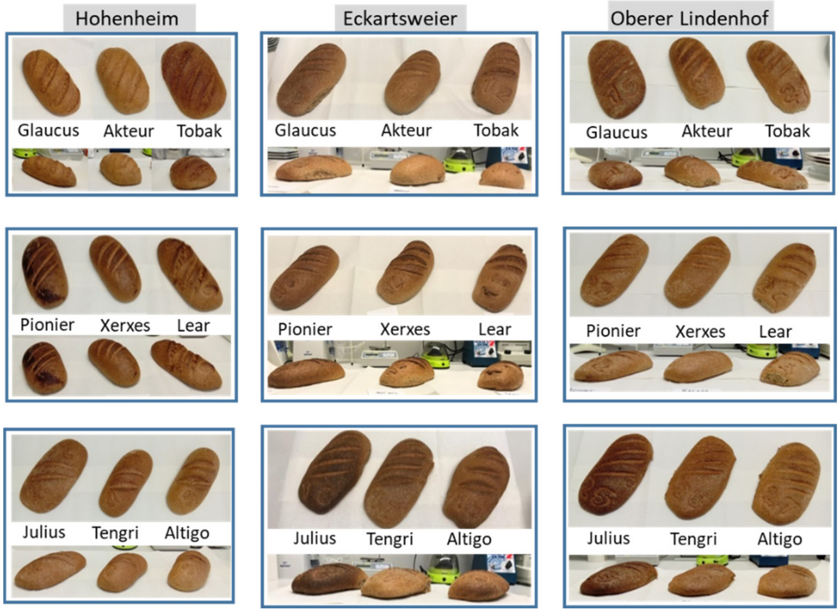

Figure S1. Bread baking. The bread was baked from each wheat cultivar for 50-60 minutes at 250 °C top heat and 230 °C bottom heat. Breads in blue rectangles were baked together in one oven.

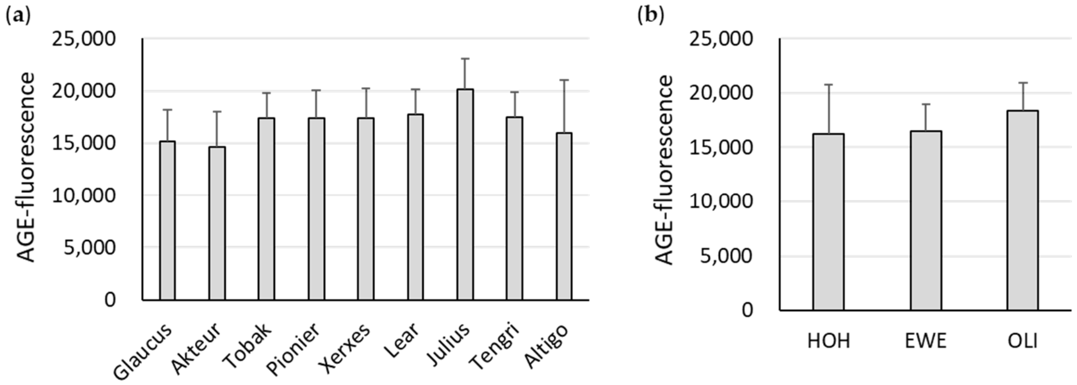

Figure S2. Wheat cultivar- and location dependence of AGE-fluorescence. **(a)** Mean AGE-fluorescence of each cultivar grown at three different places. **b)** Mean AGE-fluorescence of the different wheats grown on one place (HOH=Hohenheim; EWE=Eckartsweier; OLI=Oberer Lindenhof).

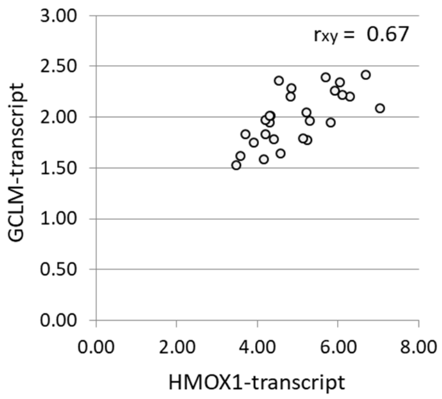

Figure S3. The association between GCLM- and HMOX1-transcript. The association between GCLM-transcript and HMOX1-transcript was determined by the Pearson correlation coefficient (r_{xy}).

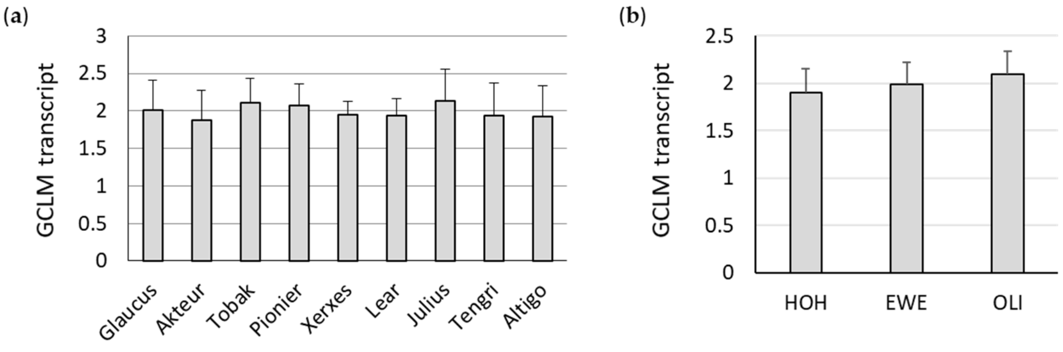

Figure S4. Wheat cultivar- and location dependence of GCLM induction. **(a)** The antioxidant potential of each cultivar grown at three different places. **b)** The mean antioxidant potential (reflected by induction of GCLM transcript) of different wheats grown on one place (HOH=Hohenheim; EWE=Eckartsweier; OLI=Oberer Lindenhof).

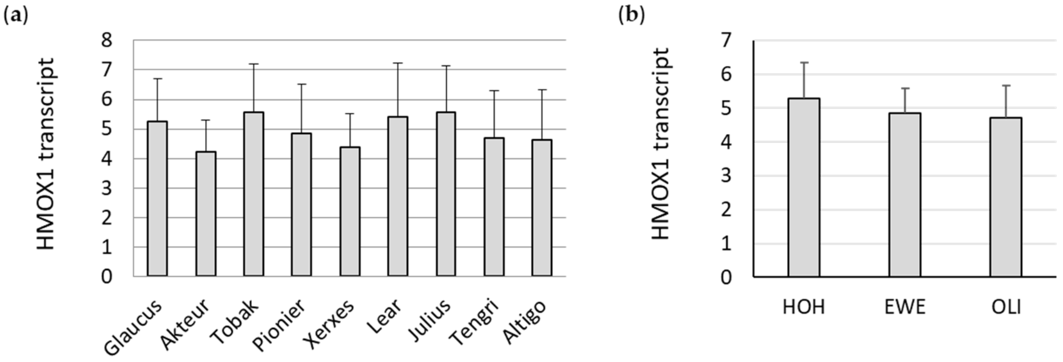

Figure S5. Wheat cultivar- and location dependence of HMOX1 induction. **(a)** The antioxidant potential of each cultivar grown at three different places. **b)** The mean antioxidant potential (reflected by the induction of HMOX1 transcript) of different wheats grown on one place (HOH=Hohenheim; EWE=Eckartsweier; OLI=Oberer Lindenhof).

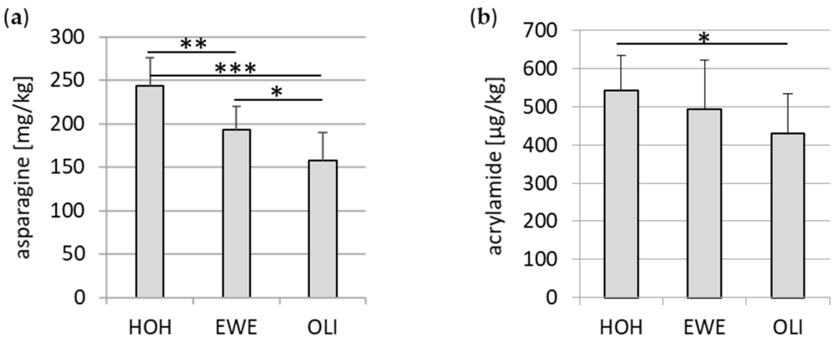

Figure S6. Location dependence of asparagine and acrylamide. Mean value of different wheats grown on one place (HOH=Hohenheim; EWE=Eckartsweier; OLI=Oberer Lindenhof) for the asparagine **(a)** and the acrylamide **(b)**. * $p<0.05$; ** $p<0.01$; *** $p<0.001$; t-test.

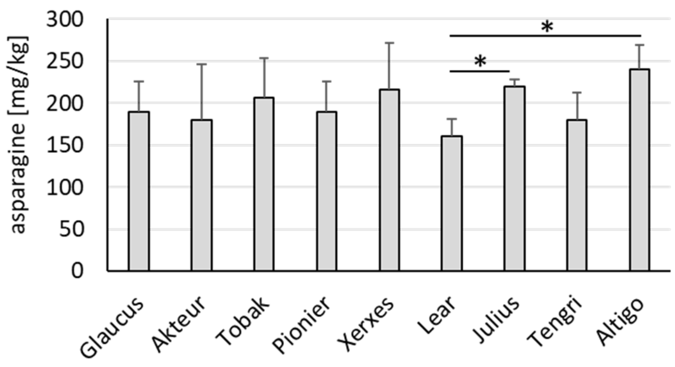

Figure S7. The mean asparagine value of each cultivar grown at three different places. * p<0.05; t-test.

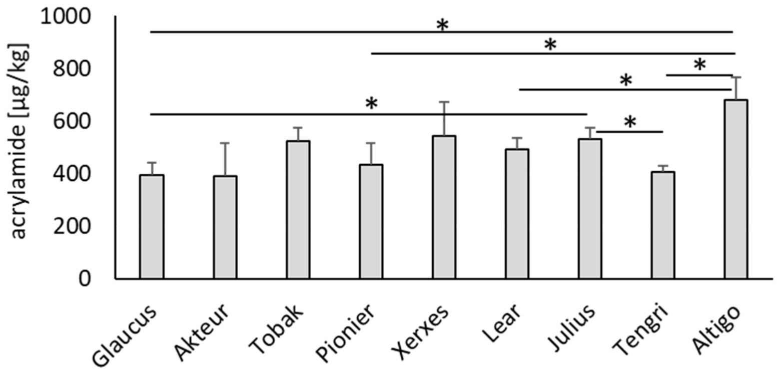

Figure S8. The mean acrylamide value of each cultivar grown at three different places. * p<0.05; t-test.

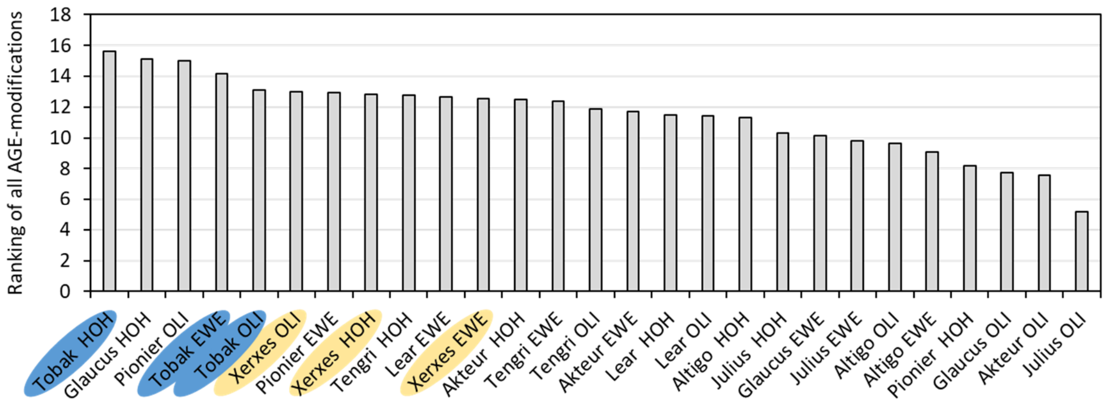

Figure S9. The mean of all ranked AGE-modifications and hexose. The wheat cultivar Tobak is highlighted in blue and Xerxes in yellow. The mounting place is indicated with HOH (Hohenheim), EWE (Eckartsweier) and OLI (Oberer Lindenhof) together with the names of the nine wheat cultivars.
